# Optimal Quantization Scheme for Data-Efficient Target Tracking via UWSNs Using Quantized Measurements

**DOI:** 10.3390/s17112565

**Published:** 2017-11-07

**Authors:** Senlin Zhang, Huayan Chen, Meiqin Liu, Qunfei Zhang

**Affiliations:** 1 State Key Laboratory of Industrial Control Technology, Hangzhou 310027, China; slzhang@zju.edu.cn; 2 College of Electrical Engineering, Zhejiang University, Hangzhou 310027, China; chenhuayan@zju.edu.cn; 3 School of Marine Science and Technology, Northwestern Polytechnical University, Xi’an 710072, China; zhangqf@nwpu.edu.cn

**Keywords:** optimal quantization, data-efficiency, target tracking, underwater wireless sensor networks

## Abstract

Target tracking is one of the broad applications of underwater wireless sensor networks (UWSNs). However, as a result of the temporal and spatial variability of acoustic channels, underwater acoustic communications suffer from an extremely limited bandwidth. In order to reduce network congestion, it is important to shorten the length of the data transmitted from local sensors to the fusion center by quantization. Although quantization can reduce bandwidth cost, it also brings about bad tracking performance as a result of information loss after quantization. To solve this problem, this paper proposes an optimal quantization-based target tracking scheme. It improves the tracking performance of low-bit quantized measurements by minimizing the additional covariance caused by quantization. The simulation demonstrates that our scheme performs much better than the conventional uniform quantization-based target tracking scheme and the increment of the data length affects our scheme only a little. Its tracking performance improves by only 4.4% from 2- to 3-bit, which means our scheme weakly depends on the number of data bits. Moreover, our scheme also weakly depends on the number of participate sensors, and it can work well in sparse sensor networks. In a 6×6×6 sensor network, compared with 4×4×4 sensor networks, the number of participant sensors increases by 334.92%, while the tracking accuracy using 1-bit quantized measurements improves by only 50.77%. Overall, our optimal quantization-based target tracking scheme can achieve the pursuit of data-efficiency, which fits the requirements of low-bandwidth UWSNs.

## 1. Introduction

As we know, more than the 70% of the earth’s surface is covered with seas and oceans. These are mysterious and charismatic to human beings because of the huge amount of precious resources that lie underwater, which are yet to be explored. Underwater wireless sensor network (UWSN) technologies are developing gradually to lead to possibilities to carry out the underwater explorations using sensors at all levels [1,2,3,4]. UWSNs are three-dimensional (3D) networks consisting of a variable number of sensors that are deployed to perform the monitoring tasks over a given region. These cooperative sensors are envisioned to enable applications for environmental monitoring, undersea exploration, disaster prevention, distributed tactical surveillance, and so forth. We address the issues of accurately and data-efficiently tracking a maneuvering target via UWSNs. UWSNs are the extending of wireless sensor networks (WSNs), which are used in terrestrial environments [5,6,7]. One of the significant differences [8] between UWSNs and WSNs is the bandwidth. Underwater sensors use acoustic waves, while terrestrial sensors use radio frequency waves. WSNs have many developed network protocols to ensure their high bit rates (in Gb/s) [9]. However, underwater acoustic communications suffer limited bandwidths due to the temporal and spatial variability of acoustic channels. A limited bandwidth leads to low bit rates, in the order of tens of Kb/s [10], which is extremely slow compared with WSNs. Therefore, in this paper, we improve the data-efficiency of UWSNs during target tracking tasks by reducing the length of data packets transmitted from local sensors to the fusion center.

In terms of target tracking, the general way to reduce the length of data is measurement quantization, which compresses complicated measurement information into several bits of data. Although quantization can shorten data length and reduce bandwidth cost, it also brings about bad tracking performance due to information loss after quantization. This paper addresses the issue of improving tracking performance of low-bit quantized measurements by optimal quantization. The general quantization method divides the measuring range into several uniform parts [11,12]. This kind of quantization method is simple and easy to implement; however, its tracking performance strongly depends on the number of data bits. In order to obtain better tracking performance of low-bit quantized measurements, local sensors should quantize their measurements according to optimal quantization thresholds.

The main contributions of this paper are twofold. First, we derive an easily updated optimal quantization threshold determination algorithm. Our algorithm can be divided into two parts: the offline part and the online part. The offline part undertakes solving the complicated optimization problem, and the online part can easily update real-time optimal quantization thresholds by combining optimal quantization factors with real-time target states. Second, on the basis of our optimal quantization algorithm, we propose a corresponding target tracking scheme. This achieves the pursuit of data-efficiency, which fits the requirements of low-bandwidth UWSNs.

The rest of the paper is organized as follows. In Section 2, we discuss the related work in the area of target tracking in UWSNs. In Section 3, we formulate the problem and introduce some propaedeutics. In Section 4, we introduce our optimal quantization-based target tracking scheme. In Section 5, we present our simulation results to verify our optimal quantization-based target tracking scheme and discuss its characteristics. Finally, in Section 6, the conclusions are made.

## 2. Related Work

Target tracking is a focused part in many military and civil fields, such as modern underwater defense systems and underwater navigation. Usually, unmanned underwater vehicles (UUVs) and submarines are intended targets to be tracked. As an emerging research interest, only a few works on target tracking via UWSNs can be found in the literature. In early works, based on a distributed particle filter, Huang et al. [13] proposed two tracking algorithms for tracking mobile targets in 2D underwater sensor networks. The results show that one tracking algorithm achieves a higher tracking accuracy, while the other achieves a dramatic reduction of the communication cost, energy cost, and tracking response time. Afterwards, Isbitiren et al. [14] presented a simple target tracking method utilizing only measurement information for 3D underwater sensor networks. On the basis of the time of arrival of the echoes from the target after transmitting acoustic pulses from the sensors, the ranges of the nodes to the target are determined, and trilateration is used to obtain the location of the target. This method tracks the target only on the basis of current measurements, which is adverse in terms of achieving a high target tracking accuracy. This results in tracking failure if too few sensors are involved, particularly in sparse networks. In order to obtain a better target tracking performance, an algorithm that combines the interacting multiple model (IMM) with the particle filter (PF) to cope with uncertainties in target maneuvers was proposed by Wang et al. [15]. Each underwater wireless sensor node composing the UWSNs is battery-powered, so the energy conservation problem is a critical issue. To realize energy-efficient target tracking, an algorithm named wake-up/sleep (WuS) that increases the energy efficiency of each sensor by using a distributed architecture was provided by Yu et al. [16]. At each time-step, WuS wakes up sensors that have an opportunity to detect the target and sleeping sensors that have no opportunity to detect the target. However, it wastes energy by employing all candidate sensors without the survival of the fittest. To solve this problem, Zhang et al. [11] studied the effect of sensor topology on the target tracking in UWSNs. They proposed a sensor selection method that selects the optimal topology by minimizing the posterior Cramer–Rao lower bound (PCRLB) of different sensor groups. This method improves the target tracking performance under the premise of employing an equal number of sensors. Moreover, Zhang et al. [17] proposed an adaptive sensor scheduling scheme that saves energy by adaptively changing the sampling interval. The sampling interval is variable according to whether the tracking accuracy is satisfactory or not at each time-step. Recently, Chen et al. [18] derived an artificial measurement-based energy-efficient filter that implements the tradeoff between the communication cost and tracking accuracy. It saves a great deal of energy while losing minor tracking accuracy or improves tracking performance with less additional energy cost. In this paper, we focus on improving the data efficiency of UWSNs by using optimal quantized measurements.

Quantizer design is also a focused issue in WSNs. Niu et al. [19] proposed two heuristic threshold design methods for localization in WSNs. Entropy-based heuristic quantization (EHQ) is an intuitive method and is only suited for binary quantization. Fishier information-based heuristic quantization (FIHQ) maximizes the Fisher information to obtain a better quantization performance. In [20], Li et al. proposed a distributed adaptive binary quantization scheme in which each individual sensor node dynamically adjusts the threshold of its quantizer on the basis of earlier transmissions from other sensor nodes. In [21], the quantizer provides the optimal quantization level that minimizes the predicted Cramer–Rao bound. Liu et al. [22] adopted the alternative conditional posterior Cramer–Rao lower bound as the optimization metric and designed the local quantizer adaptively by solving a particle-based non-linear optimization problem. In [23], a multiobjective optimization approach for an adaptive binary quantizer was designed, which jointly maximizes the Fisher information for decreasing the error on estimation and minimizes the sum of sensor transmission probabilities. An intuitive Gaussian likelihood-based quantization scheme was proposed in [24], which allocates more quantization thresholds to the interval generally with a higher probability and fewer thresholds to the less-probable interval. However, existing quantizers designed for WSNs have a common weak point. Their optimization algorithms are based on Fisher information and the posterior Cramer–Rao lower bound, whose objective function couples with target states. At each time-step, the sensors need to update their optimal quantizers by solving a complicated optimization problem. This is acceptable in developed terrestrial sensor networks. However, in severe underwater environments, common sensors usually are not qualified for such a complicated computational burden. In this paper, our optimization algorithm is based on minimizing the expectation of the additional error covariance caused by quantization. The final simplified objective function does not require knowledge of the sensor location or the target state. Thus the computational burden of solving the optimization problem can be performed offline. The real-time optimal quantization thresholds of sensors are easy to calculate on the basis of the predetermined optimal quantization factor and real-time target state.

## 3. Problem Formulation

This section formulates the problem of single target tracking via distributed UWSNs. The issues to be covered include the system model, distributed fusion architectures, state estimation with quantized measurements and the PCRLB with quantized measurements.

### 3.1. System Model

We consider the conventional target motion model, which is defined as

(1) $$X_{k}=F_{k-1}X_{k-1}+w_{k-1}$$

where $X_{k}$ denotes the target state (positions and velocities) at time *k*, $F_{k-1}$ is the state transition matrix at time k−1, and $w_{k-1}$ is the process noise with zero-mean white Gaussian distribution $N(0,Q_{k-1})$.

UWSNs consist of *N* wireless acoustic sensors floating at different seawater layers. The positions of sensors in Cartesian coordinates are denoted by $s^{i}=\left(x_{i},y_{i},z_{i}\right),i=1,\ldots N$. Sensors measure the distance to the target by transmitting acoustic pulses (ping) and calculating the time-of-arrival (ToA) of the pings and echoes.

The measurement model of the sensor $s^{i}$ at time *k* is given by

(2) $$Z_{k}^{i}=h_{k}^{i}\left(X_{k}\right)+v_{k}^{i}$$

where $h_{k}^{i}\left(X_{k}\right)$ is the measurement function, and $v_{k}^{i}$ is the measurement noise with zero-mean white Gaussian distribution $N(0,R_{k}^{i})$. The measurement function is given by

(3) $$h_{k}^{i}\left(X_{k}\right)=\sqrt{{\left(x_{k}-x_{i}\right)}^{2}+{\left(y_{k}-y_{i}\right)}^{2}+{\left(z_{k}-z_{i}\right)}^{2}}$$

where $\left(x_{k},y_{k},z_{k}\right)$ is the location of the target at time *k*. The corresponding Jacobian matrix $H_{k}^{i}$, which is a useful approximation technique from the well-known extended Kalman filter (EKF), of the measurement function $h_{k}^{i}\left(\cdot \right)$ is given by

(4) $$H_{k}^{i}\left(X_{k}\right)=\left[\begin{array}{c} \left(x_{k}-x_{i}\right)/d,0,\left(y_{k}-y_{i}\right)/d,0,\left(z_{k}-z_{i}\right)/d,0 \end{array}\right]$$

where $d=\sqrt{{\left(x_{k}-x_{i}\right)}^{2}+{\left(y_{k}-y_{i}\right)}^{2}+{\left(z_{k}-z_{i}\right)}^{2}}$ is the distance between the target and the sensor *i*.

### 3.2. Distributed Fusion Architectures with Quantized Measurements

**Figure 1:**
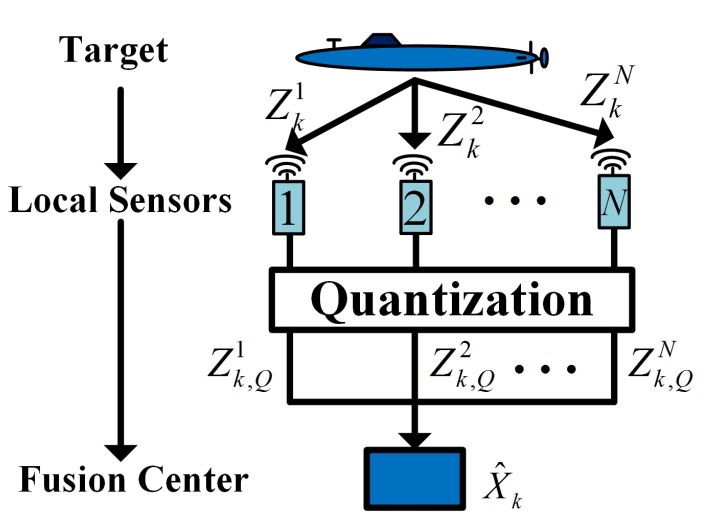
Distributed fusion architecture for target tracking with quantized measurements.

At the same time, each local sensor obtains its specific measurement about the target. The information of different sensors can be fused together to acquire more accurate estimates of target states. There are two types of fusion architectures, distributed fusion architectures and centralized fusion architectures. Distributed fusion architectures have advantages over centralized architectures in terms of lower communications and processing costs. Therefore, distributed fusion architectures are preferential for applications in resource-limited UWSNs. What is more, considering extremely low-bit-rate underwater acoustic channels, we should reduce the length of data packets sent from local sensors. This helps the fusion center to receive information from local sensors in real-time. In terms of target tracking, the general solution is measurement quantization, which compresses complicated measurement information into several bits of data. Figure 1 shows the structure of the distributed fusion system with quantized measurements. Local sensors sample the measurements ($Z_{k}^{1},Z_{k}^{2},\cdots ,Z_{k}^{N}$) from the target periodically. Because of the bandwidth limitations, local sensors quantize their measurements into finite bit measurements as ($Z_{k,Q}^{1},Z_{k,Q}^{2},\cdots ,Z_{k,Q}^{N}$). Then, local sensors transmit their quantized measurements to the fusion center. Finally, the fusion center fuses these together to obtain the fusion estimate ${\hat{X}}_{k}$.

### 3.3. State Estimation with Quantized Measurements

This section introduces how a fusion center estimates the target state ${\hat{X}}_{k}$ from quantized measurements. In general, the only point we can infer from a quantized measurement $Z_{k,Q}=i$ about the measurement before quantization $Z_{k}$ is

(5) $$\tau_{i}\leq Z_{k}<\tau_{i+1}$$

where $\tau_{i}$ and $\tau_{i+1}$ are quantization thresholds. According to this equation, Duan et al. [25] proposed an approximate minimal mean-square error (MMSE) filter algorithm to estimate the target state from quantized measurements. This assumes that target state estimate is

(6) $${\hat{X}}_{k-1}=E\left(X_{k-1}|Z_{Q}^{k-1}\right)$$

where $Z_{Q}^{k-1}=\left\{Z_{1,Q},Z_{2,Q},\cdots ,Z_{k-1,Q}\right\}$ and the corresponding error covariance

(7) $$P_{k-1}=\mathrm{Cov}\left(X_{k-1}|Z_{Q}^{k-1}\right)$$

is obtained at time k−1. According to the target motion model in Equation (1), it follows that

(8) $${\hat{X}}_{k|k-1}=F_{k-1}{\hat{X}}_{k-1}$$

(9) $$P_{k|k-1}=F_{k-1}P_{k-1}F_{k-1}^{T}+Q_{k-1}$$

Furthermore, it follows from the classical Kalman filter that

(10) $${\hat{X}}_{k}={\hat{X}}_{k|k-1}+K_{k}\left(E\left(Z_{k}|Z_{Q}^{k-1},Z_{k,Q}\right)-H_{k}{\hat{X}}_{k|k-1}\right)$$

where

(11) $$K_{k}=P_{k|k-1}H_{k}^{T}{\left(H_{k}P_{k|k-1}H_{k}^{T}+R_{k}\right)}^{-1}$$

is the Kalman gain and

(12) $$\begin{array}{cc} E(Z_{k}|Z_{Q}^{k-1},Z_{k,Q}) & =\int_{\tau_{i}}^{\tau_{i+1}}Z_{k}p\left(Z_{k}|Z_{Q}^{k-1},Z_{k,Q}\right)dZ_{k}\\ & =\frac{\int_{\tau_{i}}^{\tau_{i+1}}Z_{k}p\left(Z_{k}|Z_{Q}^{k-1}\right)dZ_{k}}{\int_{\tau_{i}}^{\tau_{i+1}}p\left(Z_{k}|Z_{Q}^{k-1}\right)dZ_{k}} \end{array}$$

Correspondingly,

(13) $$P_{k}=P_{k}^{*}+K_{k}\mathrm{Cov}\left(Z_{k}|Z_{Q}^{k-1},Z_{k,Q}\right)K_{k}^{T}$$

where

(14) $$P_{k}^{*}=P_{k|k-1}-K_{k}H_{k}P_{k|k-1}$$

and

(15) $$\begin{array}{cc} \;\mathrm{Cov}(Z_{k}|Z_{Q}^{k-1},Z_{k,Q}) & =E\left(Z_{k}Z_{k}^{T}|Z_{Q}^{k-1},Z_{k,Q}\right)-E\left(Z_{k}|Z_{Q}^{k-1},Z_{k,Q}\right)E^{T}\left(Z_{k}|Z_{Q}^{k-1},Z_{k,Q}\right)\\ & =\frac{\int_{\tau_{i}}^{\tau_{i+1}}Z_{k}Z_{k}^{T}p\left(Z_{k}|Z_{Q}^{k-1}\right)dZ_{k}}{\int_{\tau_{i}}^{\tau_{i+1}}p\left(Z_{k}|Z_{Q}^{k-1}\right)dZ_{k}}-E\left(Z_{k}|Z_{Q}^{k-1},Z_{k,Q}\right)E^{T}\left(Z_{k}|Z_{Q}^{k-1},Z_{k,Q}\right) \end{array}$$

For a detailed derivation, readers can refer to [25].

### 3.4. PCRLB with Quantized Measurements

As we know, the PCRLB provides the theoretical limit for the performance of a Bayesian estimator. For the purpose of evaluating different quantization schemes, in this section, we give an introduction about the PCRLB with quantized measurements. The PCRLB is defined as the inverse of the Fisher information matrix (FIM), and it provides a lower bound on the mean-square error (MSE) of target state estimation. There are some ways to compute the PCRLB with quantized measurements; the interested readers can refer to [26,27]. Let $p(Z_{k,Q},X_{k})$ be the joint probability density of the quantized measurement $Z_{k,Q}$ and the unknown state $X_{k}$, and let ${\hat{X}}_{k}$ be an estimate of $X_{k}$. The PCRLB on the mean-square estimation error has the form

(16) $$E\left[{\hat{X}}_{k}-X_{k}\right]{\left[{\hat{X}}_{k}-X_{k}\right]}^{T}\geq J_{k}^{-1}$$

$J_{k}$ is the 6×6 FIM with the elements

(17) $$J_{k}\left(i,j\right)=E_{p(Z_{k,Q},X_{k})}\left[-\frac{\partial^{2}\log\;\left(Z_{k,Q},X_{k}\right)}{\partial X_{k}\left(i\right)\partial X_{k}\left(j\right)}\right]$$

where $J_{k}\left(i,j\right)$ denotes the *i*th row and *j*th column element of $J_{k}$, $E_{p(Z_{k,Q},X_{k})}$ denotes the expectation with respect to $p(Z_{k,Q},X_{k})$, and $X_{k}\left(i\right)$ denotes the *i*th element of vector $X_{k}$. Let $\nabla_{X_{k}}^{X_{k}}=\nabla_{X_{k}}\nabla_{X_{k}}^{T}$ be the second-order partial derivative operator with respect to $X_{k}$. Then the above equation can be rewritten as

(18) $$J_{k}=E_{p(Z_{k,Q},X_{k})}\left[-\nabla_{X_{k}}^{X_{k}}\log\;p\left(Z_{k,Q},X_{k}\right)\right]$$

As a result of $p\left(Z_{k,Q},X_{k}\right)=p\left(Z_{k,Q}|X_{k}\right)p\left(X_{k}\right)$, $J_{k}$ can be composed into two parts:

(19) $$J_{k}=J_{k}^{D}+J_{k}^{P}$$

where

(20) $$J_{k}^{D}=E_{p\left(Z_{k,Q}|X_{k}\right)p\left(X_{k}\right)}\left[-\nabla_{X_{k}}^{X_{k}}\log\;p\left(Z_{k,Q}|X_{k}\right)\right]$$

(21) $$J_{k}^{P}=E_{p(X_{k})}\left[-\nabla_{X_{k}}^{X_{k}}\log\;p\left(X_{k}\right)\right]$$

It is noted that $J_{k}^{D}$ represents the Fisher information obtained from the quantized measurements and $J_{k}^{P}$ represents the a-priori Fisher information. We assume that there are *n* sensors and let $J_{i,k}^{D}$ denote the contribution of sensor *i* to the Fisher information. Then $J_{k}$ can be rewritten as

(22) $$J_{k}=\sum_{i=1}^{n}J_{i,k}^{D}+J_{k}^{P}$$

Because there is no closed-form solution to $J_{i,k}^{D}$ and $J_{k}^{P}$ in the above equation, particle approximation was applied to obtain the approximated solution. Predicted particles can be used to approximate $J_{i,k}^{D}$ as

(23) $$J_{i,k}^{D}\approx \frac{1}{N}\sum_{j=1}^{N}J_{i,k}^{S}\left(X_{k}^{j}\right)$$

where $X_{k}^{j}$ is the *j*th predicted particle and $J_{i,k}^{S}\left(X_{k}^{j}\right)$ is the Fisher information of sensor *i* corresponding to $X_{k}^{j}$. According to the measurement model in Equation (2), $J_{i,k}^{S}\left(X_{k}^{j}\right)$ can be given by

(24) $$\begin{array}{c} J_{i,k}^{S}=\frac{1}{2\pi R_{k}^{i}{\left[h_{k}^{i}\left(X_{k}^{j}\right)\right]}^{2}}\varphi_{k}^{i}\left(X_{k}^{j}\right)\\ \times \left[\begin{array}{cccccc} {\left(x_{i}-x_{k}^{j}\right)}^{2} & 0 & \left(x_{i}-x_{k}^{j}\right)\left(y_{i}-y_{k}^{j}\right) & 0 & \left(x_{i}-x_{k}^{j}\right)\left(z_{i}-z_{k}^{j}\right) & 0\\ 0 & 0 & 0 & 0 & 0 & 0\\ \left(x_{i}-x_{k}^{j}\right)\left(y_{i}-y_{k}^{j}\right) & 0 & {\left(y_{i}-y_{k}^{j}\right)}^{2} & 0 & \left(y_{i}-y_{k}^{j}\right)\left(z_{i}-z_{k}^{j}\right) & 0\\ 0 & 0 & 0 & 0 & 0 & 0\\ \left(x_{i}-x_{k}^{j}\right)\left(z_{i}-z_{k}^{j}\right) & 0 & \left(y_{i}-y_{k}^{j}\right)\left(z_{i}-z_{k}^{j}\right) & 0 & {\left(z_{i}-z_{k}^{j}\right)}^{2} & 0\\ 0 & 0 & 0 & 0 & 0 & 0 \end{array}\right] \end{array}$$

where $\varphi_{k}^{i}\left(X_{k}^{j}\right)$ can be calculated as

(25) $$\varphi_{k}^{i}\left(X_{k}^{j}\right)=\sum_{l=0}^{L-1}\frac{{\left\{e^{-\frac{{\left[\tau_{l}-h_{k}^{i}\left(X_{k}^{j}\right)\right]}^{2}}{2R_{k}^{i}}}-e^{-\frac{{\left[\tau_{l+1}-h_{k}^{i}\left(X_{k}^{j}\right)\right]}^{2}}{2R_{k}^{i}}}\right\}}^{2}}{p(Z_{k,Q}^{i}=l|X_{k}^{j})}$$

In order to calculate $J_{k}^{P}$, we use a Gaussian assumption such that the predicted state follows the Gaussian distribution:

(26) $$X_{k}∼N\left(\mu_{k},\Sigma_{k}\right)$$

where

(27) $$\mu_{k}=\frac{1}{N}\sum_{j=1}^{N}X_{k}^{j}$$

and

(28) $$\Sigma_{k}=\frac{1}{N}\sum_{j=1}^{N}\left(X_{k}^{j}-\mu_{k}\right){\left(X_{k}^{j}-\mu_{k}\right)}^{T}$$

Given the Gaussian assumption, it is easy to show that $J_{k}^{P}\approx \Sigma_{k}^{-1}$.

## 4. Optimal Quantization-Based Target Tracking Scheme

### 4.1. Optimal Quantization Thresholds Determination

Although quantization can shorten the data length and reduce bandwidth cost, it also brings about bad tracking performance because of information loss after quantization. Particularly for 1-bit quantized measurements, the tracking performance will be terribly bad. However, a 1-bit quantized measurement is preferential for bandwidth-limited underwater environments, if we can improve its tracking performance. Because $P_{k}$ in Equation (13) demonstrates the error covariance of a target state estimate using quantized measurements, it can be used to represent tracking performance. For simplicity, we rewrite it as

(29) $$P_{k}=P_{k}^{*}+P_{k}^{Q}$$

where $P_{k}^{Q}=K_{k}\mathrm{Cov}\left(Z_{k}|Z_{Q}^{k-1},Z_{k,Q}\right)K_{k}^{T}$. Because Equation (14) states that $P_{k}^{*}$ represents the error covariance of the target state estimate using unquantized measurements, $P_{k}^{Q}$ represents the additional error covariance caused by quantization. According to Equation (15), the quantization thresholds $\tau_{i}$ and $\tau_{i+1}$ affect the value of $P_{k}^{Q}$. This means that we can choose appropriate quantization thresholds to decrease additional error covariance and then improve target tracking performance. Supposing that the detection range of an underwater sensor is bounded to a finite interval [0,D] and that we wish to obtain a *b*-bit quantized measurement, a general quantization method is given by dividing the interval [0,D] into $L=2^{b}$ uniform parts [11,12]. Therefore, the quantization thresholds are given by the set $\tau =\{\tau_{0}=0,\tau_{1},\tau_{2},\cdots ,\tau_{L}=D\}$, and

(30) $$\tau_{i+1}-\tau_{i}=D/L$$

This kind of quantization method is simple and easy to implement. However, its tracking performance strongly depends on the number of data bits and a large *b* is needed to guarantee its tracking performance. In order to improve the target tracking performance using quantized measurements, we study the issue of optimal quantization threshold determination. According to Equation (29), we need to decrease the additional covariance $P_{k}^{Q}$ caused by quantization as much as possible. This happens by minimizing the expectation of $\mathrm{Cov}(Z_{k}|Z_{Q}^{k-1},Z_{k,Q})$:

(31) $$\tau^{opt}=\underset{\tau =\{\tau_{0},\tau_{1},\cdots ,\tau_{L}\}}{\arg\;\min}E\left(\mathrm{Cov}\left(Z_{k}|Z_{Q}^{k-1},Z_{k,Q}\right)\right)$$

Before we obtain $Z_{k,Q}$, the value of $Z_{k,Q}$ obeys the following distribution:

(32) $$P\left(Z_{k,Q}=i\right)=\int_{\tau_{i}}^{\tau_{i+1}}p\left(Z_{k}|Z_{Q}^{k-1}\right)dZ_{k}$$

On the basis of the well-known Bayesian formula, we have

(33) $$E\left(\mathrm{Cov}\left(Z_{k}|Z_{Q}^{k-1},Z_{k,Q}\right)\right)=\sum_{i=0}^{L-1}\mathrm{Cov}\left(Z_{k}|Z_{Q}^{k-1},Z_{k,Q}=i\right)P\left(Z_{k,Q}=i\right)$$

Substituting Equation (15) into Equation (33), we have

(34) $$\begin{array}{c} \;E(\mathrm{Cov}\left(Z_{k}|Z_{Q}^{k-1},Z_{k,Q}\right))\\ =\sum_{i=0}^{L-1}\int_{\tau_{i}}^{\tau_{i+1}}Z_{k}Z_{k}^{T}p\left(Z_{k}|Z_{Q}^{k-1}\right)dZ_{k}-\sum_{i=0}^{L-1}\left\{E\left(Z_{k}|Z_{Q}^{k-1},Z_{k,Q}=i\right)\times E^{T}\left(Z_{k}|Z_{Q}^{k-1},Z_{k,Q}=i\right)P\left(Z_{k,Q}=i\right)\right\}\\ =E\left(Z_{k}Z_{k}^{T}|Z_{Q}^{k-1}\right)-\sum_{i=0}^{L-1}\left\{E\left(Z_{k}|Z_{Q}^{k-1},Z_{k,Q}=i\right)\times E^{T}\left(Z_{k}|Z_{Q}^{k-1},Z_{k,Q}=i\right)P\left(Z_{k,Q}=i\right)\right\} \end{array}$$

Because $E(Z_{k}Z_{k}^{T}|Z_{Q}^{k-1})$ is independent of τ, Equation (31) is equal to

(35) $$\begin{array}{c} \tau^{opt}=\underset{\tau =\{\tau_{0},\tau_{1},\cdots ,\tau_{L}\}}{\arg\;\max}\sum_{i=0}^{L-1}\left\{E\left(Z_{k}|Z_{Q}^{k-1},Z_{k,Q}=i\right)\times E^{T}\left(Z_{k}|Z_{Q}^{k-1},Z_{k,Q}=i\right)P\left(Z_{k,Q}=i\right)\right\} \end{array}$$

For the sake of calculating $E(Z_{k}|Z_{Q}^{k-1},Z_{k,Q}=i)$, we define

(36) $$Y_{k}=\frac{Z_{k}-{\hat{Z}}_{k|k-1}}{\sqrt{S_{k}}}$$

where ${\hat{Z}}_{k|k-1}$ and $S_{k}=H_{k}P_{k|k-1}H_{k}^{T}+R_{k}$ are the mean and covariance of $p(Z_{k}|Z_{Q}^{k-1})$, respectively. This means that $p(Y_{k}|Z_{Q}^{k-1})$ obeys the standard normal distribution. Then

(37) $$\begin{array}{c} E\left(Z_{k}|Z_{Q}^{k-1},Z_{k,Q}=i\right)={\hat{Z}}_{k|k-1}+\sqrt{S_{k}}E\left(Y_{k}|Z_{Q}^{k-1},Z_{k,Q}=i\right) \end{array}$$

and

(38) $$\begin{array}{cc} E(Y_{k}|Z_{Q}^{k-1},Z_{k,Q}=i) & =\int_{m_{i}}^{m_{i+1}}Y_{k}p\left(Y_{k}|Z_{Q}^{k-1},Z_{k,Q}=i\right)dY_{k}\\ & =\frac{\int_{m_{i}}^{m_{i+1}}Y_{k}p\left(Y_{k}|Z_{Q}^{k-1}\right)dY_{k}}{\int_{m_{i}}^{m_{i+1}}p\left(Y_{k}|Z_{Q}^{k-1}\right)dY_{k}}\\ & =\frac{\int_{m_{i}}^{m_{i+1}}Y_{k}p\left(Y_{k}|Z_{Q}^{k-1}\right)dY_{k}}{P(Z_{k,Q}=i)} \end{array}$$

where

(39) $$m_{i}=\frac{\tau_{i}-{\hat{Z}}_{k|k-1}}{\sqrt{S_{k}}}$$

Because $p(Y_{k}|Z_{Q}^{k-1})$ has a standard normal distribution, we have

(40) $$\begin{array}{cc} \int_{m_{i}}^{m_{i+1}}Y_{k}p\left(Y_{k}|Z_{Q}^{k-1}\right)dY_{k} & =-\int_{p(m_{i}|Z_{Q}^{k-1})}^{p(m_{i+1}|Z_{Q}^{k-1})}dp\left(Y_{k}|Z_{Q}^{k-1}\right)\\ & =-[p\left(m_{i+1}|Z_{Q}^{k-1}\right)-p\left(m_{i}|Z_{Q}^{k-1}\right)] \end{array}$$

where $p\left(m_{i}|Z_{Q}^{k-1}\right)=1/\sqrt{2\pi }e^{-m_{i}^{2}/2}$. Substituting Equations (37), (38) and (40) into Equation (35), we have

(41) $$\begin{array}{c} \;\sum_{i=0}^{L-1}\left\{E\left(Z_{k}|Z_{Q}^{k-1},Z_{k,Q}=i\right)\times E^{T}\left(Z_{k}|Z_{Q}^{k-1},Z_{k,Q}=i\right)P\left(Z_{k,Q}=i\right)\right\}\\ =\sum_{i=0}^{L-1}\{{\hat{Z}}_{k|k-1}^{2}P\left(Z_{k,Q}=i\right)+\sum_{i=0}^{L-1}S_{k}\frac{{\left[p\left(m_{i+1}|Z_{Q}^{k-1}\right)-p\left(m_{i}|Z_{Q}^{k-1}\right)\right]}^{2}}{P(Z_{k,Q}=i)}\\ \;-2\sum_{i=0}^{L-1}{\hat{Z}}_{k|k-1}\sqrt{S_{k}}\left[p\left(m_{i+1}|Z_{Q}^{k-1}\right)-p\left(m_{i}|Z_{Q}^{k-1}\right)\right]\} \end{array}$$

We note that

(42) $$\begin{array}{c} \;\sum_{i=0}^{L-1}{\hat{Z}}_{k|k-1}\sqrt{S_{k}}\left[p\left(m_{i+1}|Z_{Q}^{k-1}\right)-p\left(m_{i}|Z_{Q}^{k-1}\right)\right]\\ ={\hat{Z}}_{k|k-1}\sqrt{S_{k}}\left[p\left(m_{L}|Z_{Q}^{k-1}\right)-p\left(m_{0}|Z_{Q}^{k-1}\right)\right]\\ ={\hat{Z}}_{k|k-1}\sqrt{S_{k}}\left[p\left(+\infty |Z_{Q}^{k-1}\right)-p\left(-\infty |Z_{Q}^{k-1}\right)\right]\\ =0 \end{array}$$

Therefore, Equation (41) can be simplified as follows:

(43) $$\begin{array}{c} \;\sum_{i=0}^{L-1}\left\{E\left(Z_{k}|Z_{Q}^{k-1},Z_{k,Q}=i\right)\times E^{T}\left(Z_{k}|Z_{Q}^{k-1},Z_{k,Q}=i\right)P\left(Z_{k,Q}=i\right)\right\}\\ ={\hat{Z}}_{k|k-1}^{2}+S_{k}\sum_{i=0}^{L-1}\frac{{\left[p\left(m_{i+1}|Z_{Q}^{k-1}\right)-p\left(m_{i}|Z_{Q}^{k-1}\right)\right]}^{2}}{P(Z_{k,Q}=i)} \end{array}$$

Furthermore, Equation (35) is equal to

(44) $$\begin{array}{c} m^{opt}=\underset{m=\{m_{0},m_{1},\cdots ,m_{L}\}}{\arg\;\max}\sum_{i=0}^{L-1}\frac{{\left[p\left(m_{i+1}|Z_{Q}^{k-1}\right)-p\left(m_{i}|Z_{Q}^{k-1}\right)\right]}^{2}}{P(Z_{k,Q}=i)} \end{array}$$

For simplicity, we define f(x) as the probability density function of the standard normal distribution. Its standard Φ-function is given by

(45) $$\Phi \left(x\right)=\int_{-\infty }^{x}1/\sqrt{2\pi }e^{-x^{2}/2}dx$$

Therefore, Equation (44) can be rewritten as

(46) $$m^{opt}=\underset{m=\{m_{0},m_{1},\cdots ,m_{L}\}}{\arg\;\max}\sum_{i=0}^{L-1}\frac{{\left[f\left(m_{i+1}\right)-f\left(m_{i}\right)\right]}^{2}}{\Phi \left(m_{i+1}\right)-\Phi \left(m_{i}\right)}$$

We define $m^{opt}$ as the optimal quantization factor, and it is independent of the target state. It is not easy to obtain an analytic solution of the above equation. However, an approximate solution for a *b*-bit quantized measurement can be provided by a numerical method. Because Equation (46) is non-linear but differentiable, the interior-point algorithm [28] is a well-suited optimization tool for solving it. Table 1 gives some useful numerical results. It should be noticed that the final simplified objective function does not require the knowledge of the sensor location or the target state. Therefore the computational burden of solving the optimization problem can be handled offline. The real-time optimal quantization thresholds of sensors are easy to calculate on the basis of a predetermined optimal quantization factor and real-time target state. According to Equation (39), optimal quantization thresholds are given by $\tau_{i}={\hat{Z}}_{k|k-1}+\sqrt{S_{k}}m_{i}$. To summarize, our quantzier design algorithm can be divided into two parts: the offline part and the online part. The offline part undertakes the solving of the complicated optimization problem, and the online part can easily update real-time optimal quantization thresholds by combining optimal quantization factors with real-time target states.

### 4.2. Optimal Quantization-Based Target Tracking Scheme

On the basis of the previous discussion, we can obtain optimal quantization thresholds. However, because of the heavy computational burden caused by integrals, it is not easy to obtain the target state and corresponding covariance according to Equations (10) and (13). To solve this problem, a PF [29] with quantized measurements is an efficient way. Here, we improve its performance by introducing our optimal quantization thresholds determination algorithm, as follows:

(1) *Particle Initialization*:

At time k=0, draw *N* particles $X_{k}^{s},s\in \left\{1,2,\cdots ,N\right\}$ from prior information of target state $p\left(X_{0}\right)∼N\left({\hat{X}}_{0},P_{0}\right)$, and set important weights as $\omega_{k}^{s}=1/N$.

(2) *Predict*:

Assume that we have already obtained the fusion estimate ${\hat{X}}_{k-1}$ and the corresponding error covariance $P_{k-1}$ at time *k*. The predicted state estimate and the corresponding error covariance at time *k* can be calculated as

(47) $${\hat{X}}_{k|k-1}=F_{k-1}{\hat{X}}_{k-1}$$

(48) $$P_{k|k-1}=F_{k-1}P_{k-1}F_{k-1}^{T}+Q_{k-1}$$

Additionally, predicted particles $X_{k|k-1}^{i}$ can be calculated as

(49) $$X_{k|k-1}^{s}=F_{k-1}X_{k-1}^{s}$$

(3) *Optimal Quantization Thresholds Determination*:

It is assumed that there are *n* sensors participating in the tracking of the target and providing the fusion center with their quantized measurements at each time-step. For certain *b*-bit quantized measurements, the corresponding optimal quantization factor is given by Table 1. Then, optimal quantization thresholds for different sensors are

(50) $$\tau_{k,i}^{j}={\hat{Z}}_{k|k-1}^{j}+m_{i}\sqrt{S_{k}^{j}},\;j\in 1,2,\cdots ,n$$

where

(51) $${\hat{Z}}_{k|k-1}^{j}=h_{k}^{j}\left({\hat{X}}_{k|k-1}\right)$$

and

(52) $$S_{k}^{j}=H_{k}^{j}P_{k|k-1}H_{k}^{jT}+R_{k}^{j}$$

According to optimal quantization thresholds, local sensors quantize their measurements and send them to the fusion center.

(4) *Importance Sampling:*

Once receiving these quantized measurements $Z_{k,Q}=\left\{Z_{k,Q}^{1},Z_{k,Q}^{2},\cdots ,Z_{k,Q}^{n}\right\}$, the fusion center fuses the measurements into a single multisensor measurement likelihood. The measurement likelihood of particle $X_{k|k-1}^{s}$ over all *n* sensors is given by

(53) $$\begin{array}{cc} p(Z_{k,Q}|X_{k|k-1}^{s}) & =\Pi_{j=1}^{n}P\left\{\tau_{k,i}^{j}\leq h_{k}^{j}\left(X_{k|k-1}^{s}\right)+v_{k}^{j}\leq \tau_{k,i+1}^{j}\right\}\\ & =\Pi_{j=1}^{n}P\left\{\tau_{k,i}^{j}-h_{k}^{j}\left(X_{k|k-1}^{s}\right)\leq v_{k}^{j}\leq \tau_{k,i+1}^{j}-h_{k}^{j}\left(X_{k|k-1}^{s}\right)\right\}\\ & =\Pi_{j=1}^{n}\left[\int_{\tau_{k,i}^{j}-h_{k}^{j}\left(X_{k|k-1}^{s}\right)}^{\tau_{k,i+1}^{j}-h_{k}^{j}\left(X_{k|k-1}^{s}\right)}\frac{1}{\sqrt{2\pi R_{k}^{j}}}e^{-t_{j}^{2}/2R_{k}^{j}}dt_{j}\right]\\ & =\Pi_{j=1}^{n}\left\{\Phi \left[\frac{\tau_{k,i+1}^{j}-h_{k}^{j}\left(X_{k|k-1}^{s}\right)}{\sqrt{R_{k}^{j}}}\right]-\Phi \left[\frac{\tau_{k,i}^{j}-h_{k}^{j}\left(X_{k|k-1}^{s}\right)}{\sqrt{R_{k}^{j}}}\right]\right\} \end{array}$$

The importance weights are updated as

(54) $$\omega_{k}^{s}=\omega_{k-1}^{s}p\left(Z_{k,Q}|X_{k|k-1}^{s}\right)$$

The importance weights are normalized as

(55) $${\bar{\omega }}_{k}^{s}=\frac{\omega_{k}^{s}}{\Sigma_{s=1}^{N}\omega_{k}^{s}}$$

(5) *Resampling:*

Multiply (suppress) $X_{k}^{s}$ with high (low) importance weights ${\bar{\omega }}_{k}^{s}$ to resample *N* particles. Then, set $\omega_{k}^{s}=1/N$.

(6) *Estimate the target state and covariance:*

The state estimate and the corresponding error covariance at time *k* can be calculated as

(56) $${\hat{X}}_{k}=\sum_{s=1}^{N}\omega_{k}^{s}X_{k|k-1}^{s}$$

(57) $$P_{k}=\sum_{s=1}^{N}\omega_{k}^{s}\left(X_{k|k-1}^{s}-{\hat{X}}_{k}\right){\left(X_{k|k-1}^{s}-{\hat{X}}_{k}\right)}^{T}$$

**Figure 2:**
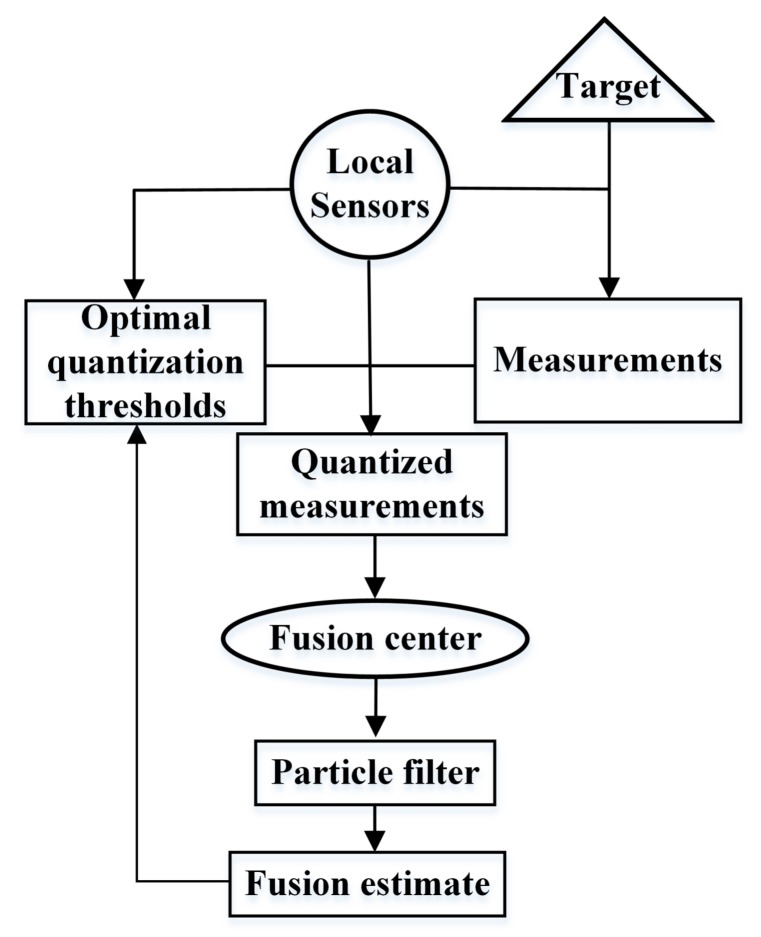
Flow chart of artificial measurements-based adaptive filter.

Finally, the fusion center broadcasts its estimate results to local sensors for the sake of determining optimal quantization thresholds at the next time-step. The flow chart of our optimal quantization-based target tracking scheme is shown in Figure 2. It shows how information flows between the fusion center and local sensors.

## 5. Simulation and Results

### 5.1. Simulation Scenario

We employed our optimal quantization-based target tracking scheme to a target tracking mission for verification. In order to obtain more realistic performance measures, the target was assumed to move in a 3D underwater environment. The monitored field was 1000 m × 1000 m × 1000 m, and the sensors were deployed as a 6×6×6 uniform grid. All local sensors were identical. Their detection radius and measure covariance were 300 m and 10 m${}^{2}$, respectively. The initial state of the target was assumed to be ${\left[300,10,300,2,10,2\right]}^{T}$. From 1 to 40 s, it moved at a *constant velocity* (CV). From 41 to 80 s, it made a coordinate turn (CT) with a turn rate of 0.052 rad/s. From 81 to 100 s, it moved at a CV. The CV and CT can be formulated as

(58) $$X_{k}=F^{\mathrm{CV}}\left(X_{k-1}\right)+w_{k}$$

(59) $$X_{k}=F^{\mathrm{CT}}\left(X_{k-1}\right)+w_{k}$$

where $F^{\mathrm{CV}}$ and $F^{\mathrm{CT}}$ are state transition matrixes, and $w_{k}$ is the process noise with zero-mean white Gaussian distributions $N(0,Q_{k})$. $F^{\mathrm{CV}}$, $F^{\mathrm{CT}}$ and $Q_{k}$ are given by

(60) $$\begin{array}{c} \begin{array}{c} F^{\mathrm{CV}}=\left[\begin{array}{cccccc} 1 & T & 0 & 0 & 0 & 0\\ 0 & 1 & 0 & 0 & 0 & 0\\ 0 & 0 & 1 & T & 0 & 0\\ 0 & 0 & 0 & 1 & 0 & 0\\ 0 & 0 & 0 & 0 & 1 & T\\ 0 & 0 & 0 & 0 & 0 & 1 \end{array}\right] \end{array} \end{array}$$

(61) $$\begin{array}{c} \begin{array}{c} F^{\mathrm{CT}}=\left[\begin{array}{cccccc} 1 & \frac{\sin(wT)}{w} & 0 & \frac{\cos(wT)-1}{w} & 0 & 0\\ 0 & \cos(wT) & 0 & -\sin(wT) & 0 & 0\\ 0 & \frac{1-\cos(wT)}{w} & 1 & \frac{\sin(wT)}{w} & 0 & 0\\ 0 & \sin(wT) & 0 & \cos(wT) & 0 & 0\\ 0 & 0 & 0 & 0 & 1 & T\\ 0 & 0 & 0 & 0 & 0 & 1 \end{array}\right] \end{array} \end{array}$$

(62) $$Q_{k}=q^{2}\left[\begin{array}{cccccc} \frac{T^{3}}{3} & \frac{T^{2}}{2} & 0 & 0 & 0 & 0\\ \frac{T^{2}}{2} & T & 0 & 0 & 0 & 0\\ 0 & 0 & \frac{T^{3}}{3} & \frac{T^{2}}{2} & 0 & 0\\ 0 & 0 & \frac{T^{2}}{2} & T & 0 & 0\\ 0 & 0 & 0 & 0 & \frac{T^{3}}{3} & \frac{T^{2}}{2}\\ 0 & 0 & 0 & 0 & \frac{T^{2}}{2} & T \end{array}\right]$$

where *q* is the intensity of the process noise. For an underwater target, we consider that only on the *xoy*-plane does it move as a CT model and that it moves as a CV model in the *Z*-axis direction.

### 5.2. Performance Verification

The simulation results were averaged over 100 Monte-Carlo runs using MATLAB. We adopted the root-mean-square error (RMSE) to assess the accuracy of target tracking and the number of packets sent from the local sensors to indicate the energy consumption.

#### 5.2.1. Performance Comparison

**Figure 3:**
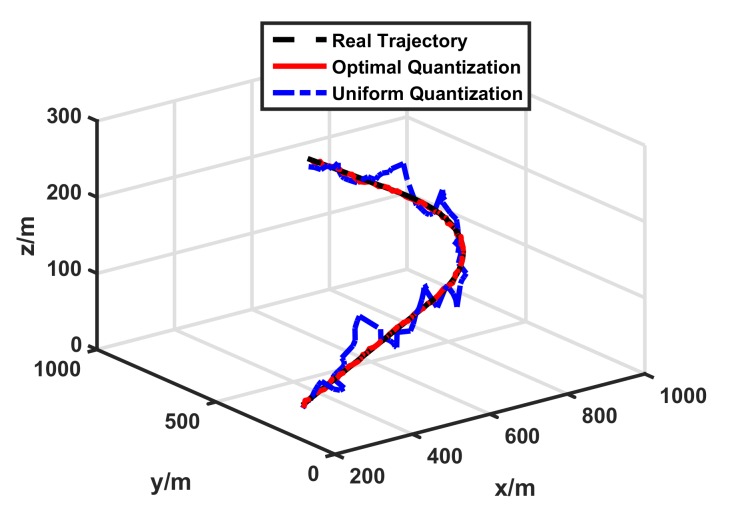
Target tracking performance with 1-bit quantized measurements.

**Figure 4:**
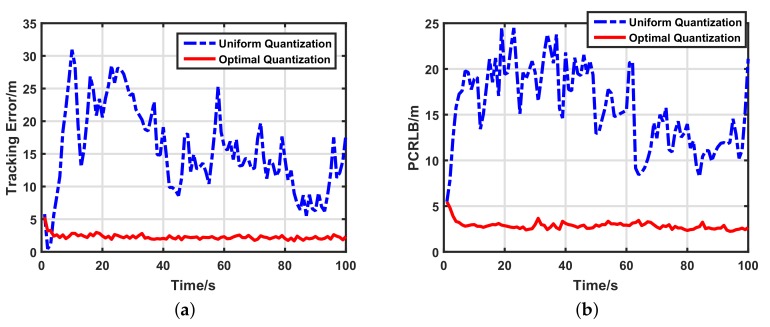
Detailed tracking performance with 1-bit quantized measurements of two schemes. (**a**) Target tracking error; (**b**) posterior Cramer–Rao lower bound (PCRLB).

In our simulation, we compared tracking performances between the conventional uniform quantization-based target tracking scheme [11,12] and our optimal quantization-based target tracking scheme. Both tracking schemes used 1-bit quantized measurements and 500 particles. Figure 3 shows the true trajectory of the target and tracking performances of different target tracking schemes. The simulation result was the same as mentioned previously. For the 1-bit quantized measurements, the tracking performance of the uniform quantization-based target tracking scheme was terribly bad. However, our optimal quantization-based target tracking scheme had a much better tracking performance, which was very close to the true trajectory. The detailed tracking error and the PCRLB of the two schemes are displayed in Figure 4. For the sake of comparison, the scalar of the PCRLB is defined as

(63) $$\zeta_{k}=\sqrt{\Omega_{k}\left(1,1\right)+\Omega_{k}\left(3,3\right)+\Omega_{k}\left(5,5\right)}$$

where

(64) $$\Omega_{k}=J_{k}^{-1}$$

Compared with the uniform quantization-based target tracking scheme, our optimal quantization-based target tracking scheme improved the tracking accuracy by about 80%. Moreover, the tracking processes of our scheme were much more stable than for the other. Thus, our scheme can remarkably improve the target tracking performance using 1-bit quantized measurements and can thus improve the data-efficiency of underwater sensors.

#### 5.2.2. Impacts of Data Lengths

**Figure 5:**
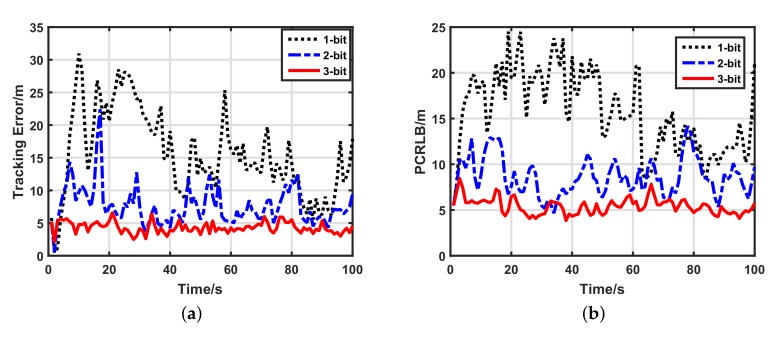
Target tracking performance of uniform quantization-based tracking scheme with different data lengths. (**a**) Target tracking error of uniform quantization-based tracking scheme; (**b**) posterior Cramer–Rao lower bound (PCRLB) of uniform quantization-based tracking scheme.

**Figure 6:**
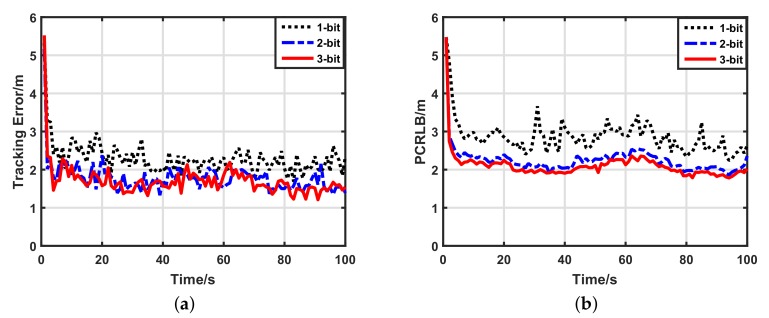
Target tracking performance of optimal quantization-based tracking scheme with different data lengths. (**a**) Target tracking error of optimal quantization-based tracking scheme; (**b**) posterior Cramer–Rao lower bound (PCRLB) of optimal quantization-based tracking scheme.

We wished to know how the number of data bits affected the target tracking performance of the two schemes. Therefore we changed this from 1-bit to 3-bit. Figure 5 illustrates the target tracking performance of the uniform quantization-based tracking scheme with a different data length. It is clear that the target tracking performance of the uniform quantization-based tracking scheme strongly depends on the number of data bits. Every bit greatly helps its tracking performance. However, as shown in Figure 6, increments of the data length changed the performance of the optimal quantization-based tracking scheme only a little. Particularly from 2- to 3-bit, the improvement caused by the data length was displayed clearly only by the PCRLB. For the sake of quantitative analysis, average tracking errors of the two schemes with a different data length are listed in Table 2. For uniform quantization, compared with the 1-bit quantized measurements, the tracking accuracy of the 3-bit quantized measurements increased by 72.54%. For optimal quantization, compared with the 1-bit quantized measurements, the tracking accuracy of the 3-bit quantized measurements increased by only 25.45%. This means that the number of data bits affects our scheme only a little. Moreover, for the 1-bit quantized measurements, compared with the uniform quantization, the optimal quantization improved the tracking accuracy by 83.4%. Thus, our scheme has good data-efficiency, which fits the requirement of low-bandwidth UWSNs very well.

#### 5.2.3. Impacts of Network Density

**Figure 7:**
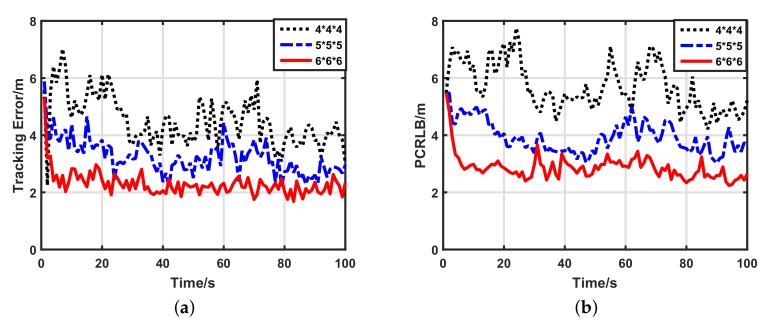
Target tracking performance of our scheme with 1-bit quantized measurements in different density networks. (**a**) Target tracking error with 1-bit quantized measurements; (**b**) posterior Cramer–Rao lower bound (PCRLB) with 1-bit quantized measurements.

**Figure 8:**
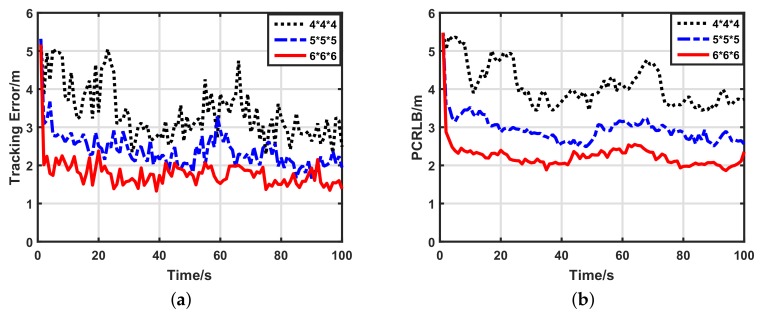
Target tracking performance of our scheme with 2-bit quantized measurements in different density networks. (**a**) Target tracking error with 2-bit quantized measurements; (**b**) posterior Cramer–Rao lower bound (PCRLB) with 2-bit quantized measurements.

**Figure 9:**
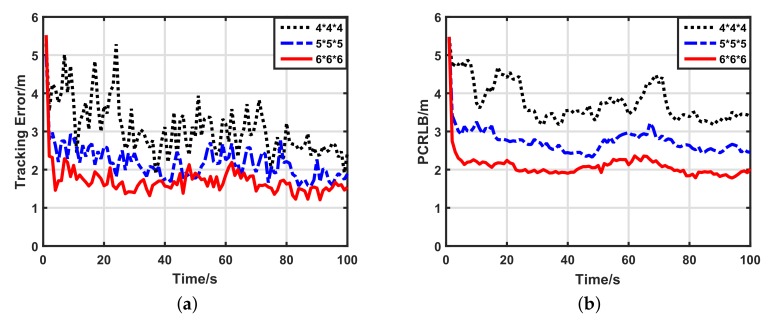
Target tracking performance of our scheme with 3-bit quantized measurements in different density networks. (**a**) Target tracking error with 3-bit quantized measurements; (**b**) posterior Cramer–Rao lower bound (PCRLB) with 3-bit quantized measurements.

Because UWSNs may be dense or sparse, we also wished to know how the network density affected the target tracking performance of our scheme. We compared the performance of three different density networks: a 6×6×6 uniform grid, a 5×5×5 uniform grid and a 4×4×4 uniform grid. Figure 7 shows the target tracking performance of our scheme using 1-bit quantized measurements in the three kinds of density networks. Clearly, in sparse networks, the tracking performance is worse than in dense networks, which is caused by the fewer number of participant sensors. Additionally, increments of the data length affect the performance of our scheme indistinctly, which is supported by Figure 8 and Figure 9. For the sake of quantitative analysis, average tracking errors and average numbers of participant sensors at each time-step in different density networks are listed in Table 3. In the 6×6×6 sensor network, compared with the 4×4×4 sensor network, the number of participant sensors increased by 334.92%, while the tracking accuracy using 1-bit quantized measurements improved by only 50.77%. Thus, our scheme weakly depends on the number of participate sensors, and it works well in sparse sensor networks. For different density networks, compared with the 1-bit quantized measurements, the 3-bit quantized measurements improved the tracking accuracy by only 31.72% (4×4×4), 32.53% (5×5×5) and 25.45% (6×6×6), respectively. Considering the low bandwidth of UWSNs, it is not worthwhile to use high-bit quantized measurements. Thus, even in different density networks, 1-bit quantized measurements are always preferential for our scheme.

## 6. Conclusions

This paper proposes an optimal quantization-based target tracking scheme. Considering the low bandwidth of UWSNs, measurement quantization is an effective way to reduce network congestion. However, conventional quantization methods lead to bad tracking performance using low-bit quantized measurements. For the pursuit of data-efficiency, we need to improve the performance of low-bit (especially 1-bit) quantized measurements. We derive an optimal quantization threshold determination algorithm, which is based on minimization of the additional covariance caused by quantization. Then, on the basis of our optimal quantization algorithm, we propose a corresponding target tracking scheme. Through simulation results, we can draw following conclusions. Firstly, our optimal quantization-based target tracking scheme performs much better than the conventional uniform quantization-based target tracking scheme. Secondly, the increment of the data length affects our scheme only a little, which means our scheme weakly depends on the number of data bits. Thirdly, our scheme works well in different density networks. Finally, our optimal quantization-based target tracking achieves the pursuit of data-efficiency, which fits the requirement of a low bandwidth of UWSNs. 

## Figures and Tables

**Table 1:** Optimal quantization factor *m* for a *b*-bit quantized measurement.

*b*-Bit Quantized Measurement	Optimal Quantization Factor *m*
b=1	m={−∞,0,∞}
b=2	m={−∞,−0.9816,0,0.9816,∞}
b=3	m={−∞,−1.6942,−0.9816,−0.4709,0,0.4709,0.9816,1.6942,∞}

**Table 2:** Average tracking errors of two schemes with different data lengths.

Quantization Methods	1-Bit	2-Bit	3-Bit
Uniform quantization	15.6724	7.6371	4.3038
Optimal quantization	2.2887	1.7847	1.7063

**Table 3:** Average tracking errors and average number of participant sensors in different density networks.

Networks	1-Bit	2-Bit	3-Bit	Participant Sensors
4×4×4	4.5077	3.3834	3.0779	5.87
5×5×5	3.2378	2.3898	2.1845	11.17
6×6×6	2.2887	1.7847	1.7063	19.66

## References

[1] Jaime L. (2013). Underwater sensor nodes and networks. Sensors.

[2] Sandeep D.N., Kumar V. (2017). Review on Clustering, Coverage and Connectivity in Underwater Wireless Sensor Networks: A Communication Techniques Perspective. IEEE Access.

[3] Liu J., Wang Z., Cui J.H., Zhou S., Yang B. (2016). A joint time synchronization and localization design for mobile underwater sensor networks. IEEE Trans. Mob. Comput..

[4] Climent S., Sanchez A., Capella J.V., Meratnia N., Serrano J.J. (2014). Underwater acoustic wireless sensor networks: Advances and future trends in physical, MAC and routing layers. Sensors.

[5] Jindal V., Verma A.K., Bawa S. (2012). Current scenario in WSNs. Int. J. Adv. Res. Comput. Eng. Technol..

[6] Calafate C.T., Lino C., Diaz-Ramire A., Cano J.C., Manzoni P. (2013). An integral model for target tracking based on the use of a WSN. Sensors.

[7] Xu N., Zhang Y., Zhang D., Zhao S., Fu W. (2017). Moving Target Tracking in Three Dimensional Space with Wireless Sensor Network. Wirel. Pers. Commun..

[8] Catipovic J. (1990). Performance limitations in underwater acoustic telemetry. IEEE J. Ocean. Eng..

[9] Felemban E., Shaikh F.K., Qureshi U.M., Sheikh A.A., Qaisar S.B. (2015). Underwater sensor network applications: A comprehensive survey. Int. J. Distrib. Sens. Netw..

[10] Akyildiz I.F., Pompili D., Melodia T. (2005). Underwater acoustic sensor networks: Research challenges. Ad Hoc Netw..

[11] Zhang Q., Liu M., Zhang S. (2015). Node Topology Effect on Target Tracking Based on UWSNs Using Quantized Measurements. IEEE Trans. Cybern..

[12] Zhou Y., Wang D., Pei T., Lan Y. (2014). Energy-Efficient Target Tracking in Wireless Sensor Networks: A Quantized Measurement Fusion Framework. Int. J. Distrib. Sensor Netw..

[13] Huang Y., Liang W., Yu H.B., Xiao Y. (2008). Target tracking based on a distributed particle filter in underwater sensor networks. Wirel. Commun. Mob. Comput..

[14] Isbitiiren G., Akan O.B. (2011). Three-dimensional underwater target tracking with acoustic sensor networks. IEEE Trans. Veh. Technol..

[15] Wang X., Xu M., Wang H. (2012). Combination of interacting multiple models with the particle filter for three-dimensional target tracking in underwater wireless sensor networks. Math. Probl. Eng..

[16] Yu C.H., Lee J.C., Choi J.W., Park M.K., Kang D.J. Energy efficient distributed interacting multiple model filter in UWSNs. Proceedings of the International Conference on Control, Automation and Systems.

[17] Zhang S., Chen H., Liu M. Adaptive sensor scheduling for target tracking in underwater wireless sensor networks. Proceedings of the 2014 International Conference on Mechatronics and Control (ICMC).

[18] Chen H., Zhang S., Liu M., Zhang Q. (2017). An Artificial Measurements-Based Adaptive Filter for Energy-Efficient Target Tracking via Underwater Wireless Sensor Networks. Sensors.

[19] Niu R., Varshney P.K. (2006). Target location estimation in sensor networks with quantized data. IEEE Trans. Signal Process..

[20] Li H., Fang J. (2007). Distributed adaptive quantization and estimation for wireless sensor networks. IEEE Signal Process. Lett..

[21] Mansouri M., Ouachani I., Snoussi H., Richard C. Cramer-Rao Bound-based adaptive quantization for target tracking in wireless sensor networks. Proceedings of the 2009 IEEE/SP 15th Workshop on Statistical Signal Processing.

[22] Liu S., Masazade E., Shen X., Varshney P.K. Adaptive non-myopic quantizer design for target tracking in wireless sensor networks. Proceedings of the 2013 Asilomar Conference on Signals, Systems and Computers.

[23] Kose A., Masazade E. A multiobjective optimization approach for adaptive binary quantizer design for target tracking in wireless sensor networks. Proceedings of the 2015 IEEE International Conference on Multisensor Fusion and Integration for Intelligent Systems (MFI).

[24] Feng X., Pan F., Gao Q., Li W. Gaussian likelihood based Bernoulli particle filter for non-uniformly quantized interval measurement. Proceedings of the 2016 19th International Conference on Information Fusion (FUSION).

[25] Duan Z., Jilkov V.P., Li X.R. State estimation with quantized measurements: Approximate MMSE approach. Proceedings of the 2008 11th International Conference on Information Fusion (FUSION).

[26] Masazade E., Niu R., Varshney P.K. (2012). Dynamic bit allocation for object tracking in wireless sensor networks. IEEE Trans. Signal Process..

[27] Cao N., Choi S., Masazade E., Varshney P.K. (2016). Sensor selection for target tracking in wireless sensor networks with uncertainty. IEEE Trans. Signal Process..

[28] Boyd S., Vandenberghe L. (2004). Convex Optimization.

[29] Ruan Y., Willett P., Marrs A., Palmieri F., Marano S. (2008). Practical fusion of quantized measurements via particle filtering. IEEE Trans. Aerosp. Electron. Syst..

